# Social Media, Body Image and Resistance Training: Creating the Perfect ‘Me’ with Dietary Supplements, Anabolic Steroids and SARM’s

**DOI:** 10.1186/s40798-021-00371-1

**Published:** 2021-11-10

**Authors:** Luuk Hilkens, Maarten Cruyff, Liesbeth Woertman, Jeroen Benjamins, Catharine Evers

**Affiliations:** 1 School of Sport and Exercise, HAN University of Applied Sciences, Nijmegen, The Netherlands; 2 Department of Methodology and Statistics, Utrecht University, Utrecht, The Netherlands; 3 Department of Clinical Psychology, Utrecht University, Utrecht, The Netherlands; 4 Department of Social, Health, and Organizational Psychology, Utrecht University, PO Box 80140, 3508 TC Utrecht, The Netherlands; 5 Department of Experimental Psychology, Helmholtz Institute, Utrecht University, Utrecht, The Netherlands

**Keywords:** Resistance training, Social media, Image-centric social media use, Body image, Androgenic–anabolic steroids, Selective androgen receptor modulators, SARM’s, Dietary supplements, Gym users

## Abstract

**Background:**

Few studies have assessed the use of dietary supplements, anabolic androgenic steroids (AAS) and selective androgen receptor modulators (SARM) in male gym users. The comparison of physical appearance with others on social media and the exposure to fitness-related content on social media (i.e., image-centric social media use) may have a profound role in using these compounds due to its role in creating negative body images in male gym users.

**Objective:**

Provide contemporary data on the use of dietary supplements, AAS and SARM among young male gym users, and test the hypothesis that social media is associated with the use of dietary supplements, AAS and SARM, as a result of a negative body image.

**Methods:**

In this cross-sectional study, conducted in the Netherlands, male gym users (*N* = 2269; 24 ± 6 years) completed an online questionnaire including self-reported measures regarding resistance training participation, image-centric social media use, dietary supplement intake, and body image. The prevalence of AAS and SARM use was assessed with randomized response, a technique to ask sensitive questions indirectly.

**Results:**

Of all participants, 83% used ergogenic dietary supplements (mainly protein and creatine), and an estimated 9 versus 2.7% had ever used AAS versus SARM. Image-centric social media use was positively associated with the use of dietary supplements (*r* = .26; *p* < 0.01) and AAS (*p* < 0.05), but not SARM. Image-centric social media use was associated with a more dissatisfied body image (*r* = .34; *p* < 0.01). Body image did not mediate the relationship between image-centric social media use and the use of doping compounds.

**Conclusions:**

The use of dietary supplements in young male gym users is exorbitant, with the use of AAS and SARM being substantial. Image-centric social media use is positively associated with the use of dietary supplements and AAS.

**Supplementary Information:**

The online version contains supplementary material available at 10.1186/s40798-021-00371-1.

## Key Points

- This novel study was the first to report a lifetime prevalence rate of SARM use (2.7%) among a sample of young male gym users. Lifetime versus current prevalence rates of AAS use was 9.0 versus 3.6%, and a high prevalence of dietary supplements such as protein (81.2%), creatine (46.2%) and pre-workout (44.2%) was observed.
- The combination of exposure to fitness-related content on social media and comparing one’s physical appearance with others based on this fitness-related content was associated with the use of dietary supplements and AAS.
- Considering the significant use of dietary supplements, AAS, and SARM among young male gym users, fitness associations, anti-doping agencies and sports medicine communities should work together, to support gym users to reach their fitness goals in healthy and sustainable manners.

## Introduction

Resistance training by recreational sportspeople in fitness settings has become increasingly popular worldwide [1]. To illustrate, in 2019, fitness was the most popular sport in the Netherlands, with ~ 3.2 million citizens registered as a gym club member, which is almost 20% of the national sample [2]. Besides health-related reasons, improving physical appearance by increasing muscle mass and decreasing body fat is a predominant motive for engaging in resistance training in fitness settings [3, 4]. However, even with much training and self-discipline, gaining muscle mass and reaching a muscular and toned body is a slow process that takes effort, time, and patience.

Dietary supplements are believed to accelerate the process of reaching the desired muscular body. Indeed, a high use of ‘muscle-building’ supplements as protein shakes, pre-workout supplements and creatine among recreational gym users has been observed [3, 5, 6]. To illustrate, 36.8% of the gym members in Brazil report the regular use of supplements, such as protein and creatine, mainly to increase strength and muscle mass [3]. In reality, however, protein and creatine provide only marginal additional gains in muscle mass with resistance training [7, 8].

In their quest for fast or unrealistic results, some gym users are also attracted to other, more active compounds than dietary supplements, such as anabolic androgenic steroids (AAS) [6, 9, 10], that result in fast and significant increases in muscle mass [11]. Although highly effective, using AAS can have severe negative health consequences, including an increased risk of cardiovascular incidents [12–15]. In addition to AAS, there are some indications, mainly based on analysis of online sales of doping substances [16], that gym users also started using more experimental drugs, such as selective androgen receptor modulators (SARM) [17, 18]. However, little is known about the efficacy and safety of the use of SARM in humans [19]. Regardless, both SARM and AAS, often termed together as ‘image and performance-enhancing drugs,’ are predominantly used to increase muscularity and modify appearance [9, 10, 18]. Particularly the group of young male gym users has been indicated as ‘at risk’ for the use of image and performance-enhancing drugs [20]. A recent meta-analysis indeed showed higher prevalence rates in gym users (~ 18.4%), compared to the prevalence rate in the overall global population (~ 3.3%) [21]. Surprisingly, the most recent data from the Netherlands showed a prevalence rate of AAS use among gym users of only 1.0% [4], although these data date back to 2013. In contrast to AAS, there are currently no prevalence data available on the use of SARM. Also, it must be noted that research on the prevalence of AAS or other doping use may be substantially flawed, as this type of research is highly sensitive for social desirability bias [4, 21, 22]. Taken together, this highlights the need for up to date, high-quality research on image and performance-enhancing drug use among male gym users.

Although data among gym users are limited, it is generally believed that gym users mainly consult the internet and social media for information regarding training, nutrition and supplements [3]. Problematic, though, is that social media is not only a seedbed for misinformation [23], there is also a growing body of evidence showing that social media use is associated with body image concerns and other negative health outcomes, such as eating disorders [24, 25]. Possible explanations why social media may cause body dissatisfaction include the continuous physical appearance comparisons with idealized images of models and peers [26, 27]. Indeed, social media typically depicts illustrations of ‘perfect’ male bodies, referring to muscular and toned bodies [26]. Besides body image, exercise and nutritional behavior of gym users may also be affected by exposure to fitness-related content on social media, such as images of physically fit peers or fitness influencers performing resistance exercise, promoting dietary supplements, or advocating a ‘bodybuilding lifestyle’ [28]. Striving for these perfectly depicted bodies, conversely, can have undesirable health implications regarding body image and eating disorders [25, 29, 30].

Although most of the research on social media and body image has focused on women [26], evidence is emerging that social media also plays an important role in body image concerns in young men, as the drive of young men for a bigger, more muscular body is becoming more prevalent [31, 32]. In light of striving toward an idealized body, a negative body image may be an important factor in explaining why social media may be associated with behaviors aimed at accelerating the desired muscular body in young male gym users, such as using dietary supplements and image and performance-enhancing drugs [33, 34]. This raises the question if and to what extent the comparison of physical appearance with others on social media in combination with the exposure to fitness-related content on social media, labelled in the current study as *image-centric social media use*, is associated with the use of image and performance-enhancing drugs, specifically in young male gym users.

### The Present Study

Firstly, the current study aimed to provide contemporary data on the use of ergogenic dietary supplements, AAS, and SARM among young male gym users, with the prevalence of AAS and SARM use investigated with randomized response measures, a technique to omit social desirability and response bias. Next, as so far no direct evidence exists, the current study also aimed to provide a first empirical test of the idea that image-centric social media use, is associated with the use of these compounds in young men as a result of a negative body image. To this end, the prevalence regarding the use of dietary supplements, AAS, and SARM was assessed in a large cohort of young male gym users in the Netherlands, in combination with data on social media use and body image. In case social media use indeed predicts the use of dietary supplements, AAS, and SARM, mediation analyses will be conducted to test whether these effects are explained by a negative body image. We hypothesized that image-centric social media exposure is positively associated with the use of dietary supplements, AAS and SARM and that this relationship is mediated by body image.

## Methods

### Participants

In total, 2269 male participants completed the study. Participants had a mean age of 24 (SD = 6, range 18–40) years, and the majority of participants reported Dutch as their nationality (97%). All participants were gym users and performed resistance training on average for 7 h/ week (SD = 3), for on average 5 years (SD = 5). See Additional file 1 for other characteristics.

### Procedure

To reach a national representative population of gym users, flyers were distributed via fitness clubs, social media and online fora that were related to fitness, resistance training, nutrition and supplements across the Netherlands. Additionally, fitness clubs registered in the trade register of the Netherlands Chamber of Commerce were randomly selected. At least two attempts were made to contact the owners of these fitness clubs by telephone, to ask to share the study advertisement with their club members. In addition, national influencers on social media were asked to share the advertisement, and a nationwide social media advertisement campaign was launched. Data collection took place in September 2020, using a web-based survey designed with Qualtrics™. In this period, gyms had been reopened for ~ 8 weeks after having been closed for ~ 14 weeks due to Covid-19-related lockdown.

The advertisement included a link to the online study. After reading the information letter and giving informed consent, three questions assessed whether participants met the inclusion criteria. Inclusion criteria were being male, being between 18 and 40 years old, and performing resistance training. Only if all three inclusion criteria were met, participants were included and forwarded to the remainder of the study. After assessing demographics and details about their resistance training routine, participants answered questions regarding social media use, body image and the use of dietary supplements and image and performance-enhancing drugs (see below). The full original questionnaire is available via the Open Science Framework (https://osf.io/4kcy7/). After completion, participants were thanked and among interested participants there was a raffle to obtain gifts such as certificates, protein powder or fitness-related books. The study protocol was reviewed and approved by the Faculty Ethical Committee of Utrecht University (#20-129).

### Measures

#### Demographics

We asked for age, gender, nationality, education level, body weight and height.

#### Resistance Training

After explaining what was considered as resistance training (training with weights, such as barbells, dumbbells or machines), open ended questions assessed how many years and how many hours per week participants were involved in resistance training. Further, participants had to indicate the main reasons to partake in resistance training [4], the specific type of training (e.g., resistance training in a fitness setting, powerlifting, weightlifting), and to what extent fitness was important to them on a scale ranging from 1 (very unimportant) to 5 (very important). Finally, they were asked to indicate whether they participated in bodybuilding competitions (yes/no).

#### Social Media Use

Measurement of social media use was based on research by Fardouly and coworkers [35] and divided between *frequency of social media use* and *image-centric social media use*.

To measure frequency of social media use, participants were asked to rate on 7-point scales to what extent they used the social media platforms Facebook, Instagram, Snapchat, YouTube or ‘other’ ranging from 1 (not at all) to 7 (very often). They also rated on a 8-point scale, ranging from 1 (not at all) to 8 (every 5 min), how often they checked their social media, and on a 13-point scale ranging from 1 (5 min or less) to 13 (10 h or more) how much time they spent on social media on a typical day. A principal components analysis was performed for the construction of the frequency scale (Cronbach’s Alpha = 0.76). See Additional file 4 for a description of the construction (principal component analysis) of the frequency of social media use scale.

To measure ‘image-centric social media use’ (in figures and tables abbreviated as ‘ISMU’), we created a novel measure. Image-centric social media use refers to the combination of the exposure to fitness-related content on social media and comparing one’s physical appearance with others based on this fitness-related social media content. Before assessing image-centric social media use, participants were first informed that ‘fitspiration’ refers to a combination of fitness and inspiration and that it contains images related to, for example, gym users or bodybuilders showing their muscles. We used the more generally known term ‘fitspiration,’ rather than ‘image-centric social media use’ to not burden the participants with professional terms. Eight items included the amount of contact with fitness-related content and the comparison of the participants’ physical appearance with peers and so-called influencers, with the latter being mainly based on the Physical Appearance Comparison Scale (PACS) modified by Fardouly and Vartanian [36]. Participants were first asked on 5-point scales how often they look at images depicting gym users (peers or models) or bodybuilders showing their muscles on social media, ranging from 1 (never) to 5 (always). Then, they rated on 5-point scales ranging from 1 (never) to 5 (very often): ‘When looking at photos of the following people on social media, how often do you compare your physical appearance to theirs?’ The target groups included either family, friends, acquaintances (i.e., people they know personally) or celebrities such as fitness models, actors and athletes. Participants also indicated their proportion of time on social media devoted to fitness, bodybuilding and supplements on scales ranging from 1 (a few) to 5 (very much). Finally, participants indicated on four items, on 5-point scales ranging from 1 (very unimportant) to 5 (very important), the extent to which they deemed it important to (a) appear physically well with high muscularity and low body fat and (b) how other people judge their physical appearance, and the extent to which (c) fitness models and fitness influencers versus (d) friends and acquaintances were important for how they appreciated their own bodies. A principal components analysis was used to obtain the image-centric social media use scale, with scale scoring containing a weighted average of the item scores, with weights proportional to the component loadings of the 1st principal component. The scale scores were standardized with mean 0 and variance 1. The component explained 42.4% of the variance of the items, and the reliability analysis of the items yielded a Cronbach’s Alpha of 0.76. See Additional file 4 for a description of the construction (principal component analysis) of the image-centric social media use scale.

#### Body Image

Body image was assessed by the Revised Male Body Attitudes Scale (MBAS-R [37]), including 15 questions. Participants rated on 5-point scales (1 = never to 5 = always) how satisfied they were with their muscularity (7 questions; e.g., ‘I think I have too little muscle on my body’), their body fat (5 questions; e.g., ‘I think I have too much fat on my body’) and their height (3 questions; e.g., ‘I feel ashamed of my height’). The Cronbach’s alphas for the subscales were *α*_muscularity_ = 0.84, *α*_body fat_ = 0.88, *α*_height_ = 0.72, and for the overall body image scale *α* = 0.79. In the context of this paper we report the overall body image scale and the subscale muscularity as descriptive data, and we used the overall body image scale for the mediation analyses. For the descriptive data, reporting an average score above 3.5 was considered as an *unsatisfied* body image, which relates to standardized scores of 1.65 above the mean.

#### Dietary Supplements and Pre-workout

Supplements were described as sports nutritional supplements like protein powders, shakes and bars, weight gainers, and performance enhancing supplements such as creatine, caffeine, beta-alanine, sodium bicarbonate or pre-workout. It was stressed that (multi)vitamin preparations and omega-3 fatty acids were *not* considered as supplements. Participants first rated if they had been using supplements the preceding four weeks (yes/no). If so, questions regarding the prevalence and motives for the use of dietary supplements were assessed, based on work by Wardenaar and colleagues [5]. Participants had to indicate their motives for supplement use (e.g., increasing muscle mass, increasing muscle strength, decreasing fat mass, more energy to exercise). Next, participants indicated to what extent they consulted the following sources of information regarding using supplements: social media, websites, personal trainer/fitness instructor, sports dietician/nutritionist, magazines, scientific literature, books, family/friends/sport buddies or something else. Next, they rated if they had been using pre-workout supplements the preceding four weeks (yes/no). If so, the frequency was assessed: participants had to imagine they do 7 strength training session per week and had to indicate how many of those workouts they use a pre-workout supplement. The most important motive for using pre-workout supplements was assessed by an open-entry question. Then, the prevalence of supplement use was rated on a 31-point scale (0 = zero days per month, 30 = every day a month). The following 11 dietary supplements where included in the questionnaire: protein, branched-chain amino acids, weight gainer (carbohydrates), fat burners, creatine, beta-alanine, caffeine, sodium bicarbonate, beta-hydroxy-beta-methylbutyrate (HMB), collagen, citrulline and the option ‘other.’ Finally, participants rated the extent to which using supplements was important for them on a scale ranging from 1 (very unimportant) to 7 (very important). See Additional file 4 for a description of the construction (principal component analysis) of the dietary supplement use scale.

#### Image and Performance-Enhancing Drugs

The use of AAS and SARM was assessed using the randomized response technique (RRT) [38], specifically, an adapted version of the Kuk’s design was used [39]. The RRT guarantees the anonymity of respondents when answering sensitive questions, such as the use of doping. The basic idea of the technique is to randomly perturb the answers to the sensitive question with probabilities set by the researcher. These perturbations offer privacy protection, since the answer does not necessarily coincide with the status on the sensitive attribute. But because the perturbation probabilities are known, a prevalence estimate of the sensitive attribute can be made on the group-level [38]. A meta-analysis showed that this technique is a more valid option when compared to direct questions, when researching sensitive issues [40]. Accordingly, the RRT has previously been used effectively in research evaluating the prevalence of doping use in elite athletes [22] and regular gym users [4]. For a full description of the method used in the current study, see Additional file 2.

The three sensitive questions posed in the RRT-format were: (1) whether participants used AAS in the last year; (2) whether they had used AAS at any point during their life; and (3) whether they had used SARM at any point during their life. During the questionnaire, participants were provided with the (brand) names of the anabolic steroids commonly used in the Netherlands (e.g. testosterone, nandrolone (‘Deca’), trenbolone (‘tren’), Stanozolol (‘Winstrol’) and oxandrolone (‘Anavar’)) [12].

### Data Analysis

Statistical analyses and calculation were performed using SPSS 25.0 (IBM Corp., Armonk, NY) and R 4.0.5 [41]. The R package ‘psych’ [42] was used for the principal components and reliability analysis. Associations between variables were analyzed using Pearson’s correlation coefficient. For each mediation analysis, four regression analyses were conducted to assess the total, direct and indirect effects of the predictor image-centric social media use on each of the dependent variables: supplements, AAS (current and lifetime use) and SARM (lifetime use). The effects were tested using directional hypotheses with one-sided t-tests. For the latter three dependent variables, logistic regression models were used that account for randomized response [43]. For an extensive description of the mediation analyses on social media use, dietary supplements, body image and AAS and SARM use, see Additional file 3. Data are presented as mean (SD) and percentages (%).

## Results

### Resistance Training

On average, participants were involved in resistance training for 5 years (SD = 5) and trained on average 7 h per week (SD = 3). The type of training reported by the majority of the participants was regular strength training in a gym/ recreational bodybuilding (44.6%). For the remainder, participants were mainly engaged in CrossFit (4.1%), powerlifting (3.6%) or Olympic weightlifting (2.2%), but most of the time these sports were combined with regular strength training in a gym/ recreational bodybuilding (31.7%). Only 2.4% of the population reported to participate in bodybuilding competitions. Reported motives to perform resistance training were: improving physical appearance by increasing muscle mass and decreasing fat mass (45.6%), improving overall health (23.3%), increasing muscle strength (17.0%), enhance sports performance (11.2%) or other reasons (3.0%). Of all participants, 78.7% rated resistance training as a (very) important component of their daily life.

### Frequency of Social Media

Of the sample, 80 and 95% reported to have a Facebook and Instagram account, respectively. The most frequently used social media platforms were Instagram (‘often’ to ‘very often’: 74.6%) and YouTube (‘often’ to ‘very often’: 66.1%), followed by Snapchat (‘often’ to ‘very often’: 45.2%), Facebook (‘often’ to ‘very often’: 18.9%), and other (‘often’ to ‘very often’: 11.2%). Most participants (72.8%) checked their social media every few hours to every hour, with 10.9% checking their social media every 30 min or more. The majority of the participants (56.2%) spent 1–2 h a day on social media, with 12.3% spending 3 h a day or more on social media.

### Image-Centric Social Media

While the total score of the image-centric social media scale was used for mediation analysis, we report here the descriptives of some single items included in the total scale to provide a compelling illustration. Related to watching fitness-related content on social media, 32.9% of all participants indicated that they ‘very often’ to ‘always’ watched people and bodybuilders workout and show their muscles. In addition, 37.3% indicated that they watched ‘much’ to ‘very much’ content related to fitness, bodybuilding and supplements. Related to physical appearance comparison on social media, of all participants, 71.5% indicated that it is ‘important’ to ‘very important’ to have a good physical appearance (muscular and/or low body fat). Also, 30.1% reported that it is ‘important’ to ‘very important’ what others think of the participants’ physical appearance. Furthermore, 27.7% indicated that people they personally know (e.g., friends and family) are ‘important’ to ‘very important’ in the way they look at their own physique, as opposed to 10.5% of people they do not personally know (e.g., fitness models, actors and athletes).

### Body Image

**Fig. 1:**
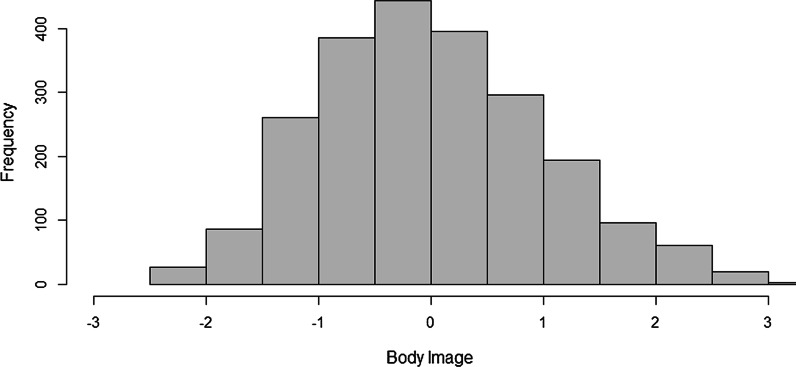
Histogram of standardized scores on body image (BI) as measured in young male gym users with the Revised Male Body Attitudes Scale (MBAS-R). Scores of − 1 representing 1 SD below the mean and + 1 representing 1 SD above the mean

Of all participants, 5.1% were not satisfied with their body (i.e., average score above 3.5 of the 5 point scale, which corresponds to standardized score of 1.65 above the mean), and 19.6% was not satisfied with their muscularity. See Fig. 1 for the distribution of the body image scores on the MBAS.

### Prevalence of Dietary Supplement and Pre-workout Use

**Fig. 2:**
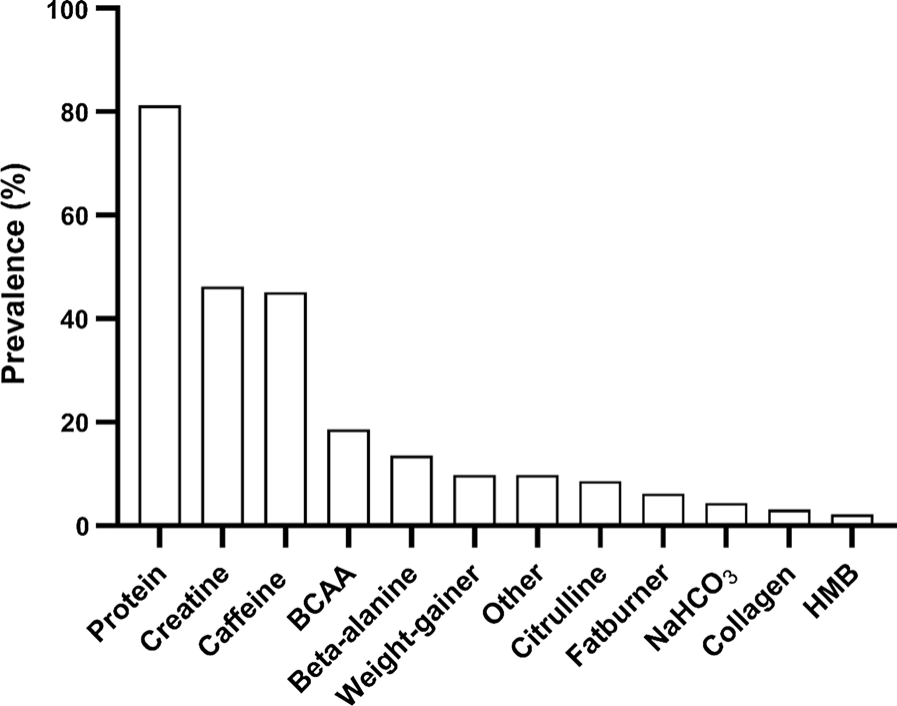
Prevalence (%) of dietary supplement use in young male gym users in the Netherlands. BCAA: branch-chained amino acids; NaHCO_3_: sodium bicarbonate; HMB: beta-hydroxy-beta-methylbutyrate

The prevalence of the use of the different dietary supplements is presented in Fig. 2. Of all participants, 82.8% reported to have used dietary supplements in the last four weeks. The most reported supplements were protein (81.2%), creatine (46.2%) and caffeine (45.1%). Of all participants, 25% vs. 29.6% of the participants indicated *daily use* of protein versus creatine, respectively. The most reported motives for the use of supplements were to increase muscle mass (53.4%), to increase the energy to train (22.4%), to increase muscular strength (21.8%), or to decrease fat mass (2.4%). Of the participants using supplements, 44.2% reported to have used a pre-workout formula in the past 4 weeks, with 25.4% using pre-workout every training day, mainly to stimulate ‘motivation,’ ‘focus,’ ‘concentration,’ and the ‘energy boost.’ The main sources of information regarding the use of supplements were websites (‘often’ to ‘very often’: 65.4%), scientific literature (‘often’ to ‘very often’: 43.6%), friends and family (‘often’ to ‘very often’: 41.7%), social media (‘often’ to ‘very often’: 32%), fitness trainer/ personal trainer (‘often’ to ‘very often’: 26.3), sports dietitian of sports nutritionist (‘often’ to ‘very often’: 16.2%), books (‘often’ to ‘very often’: 16.5%) or magazines (‘often’ to ‘very often’: 5.6%). Of all participants, 27.2% rated the use of supplements to be ‘important’ to ‘very important.’

### Prevalence of Image and Performance-Enhancing Drug Use

The prevalence of AAS and SARM use is presented in Table 1.

**Table 1:** Prevalence of AAS and SARM use in young male gym users in the Netherlands

Question	Prevalence (% and 95% CI)
Have you ever used AAS?	9.0 (6.4–11.6)
Have you used AAS in the last 12 months?	3.6 (1.2–6.1)
Have you ever used SARM?	2.7 (0.3–5.1)

AAS: androgenic anabolic steroids; SARM: selective androgen receptor modulator

### Main Analyses

Table 2 contains the correlation coefficients for image-centric social media use, frequency of social media use, supplement use and body image (total scale and muscularity subscale). Noteworthy is that image-centric social media use showed significant associations with supplement use (*r* = 0.26; *p* =  < 0.01) and body image (*r* = 0.34; *p* = < 0.01), while the frequency of social media use refrained from significant pairings.

**Table 2:** Pearson correlations between image-centric social media use (ISMU), frequency of social media use (FSMU), supplement use (SUPP), and body image (split out for body image total scale and muscularity subscale), among young male gym users in the Netherlands

	ISMU	FSMU	SUPP	Body image_total scale_	Body image_muscularity subscale_
ISMU	–				
FSMU	.23*	–			
SUPP	.26**	.09	–		
Body image_total scale_	.34**	.13	.09	–	
Body image_muscularity subscale_	.30**	.15	.15*	.70**	–

Correlation is significant at the * 0.05 level or ** 0.01 level (2-tailed)

**Fig. 3:**
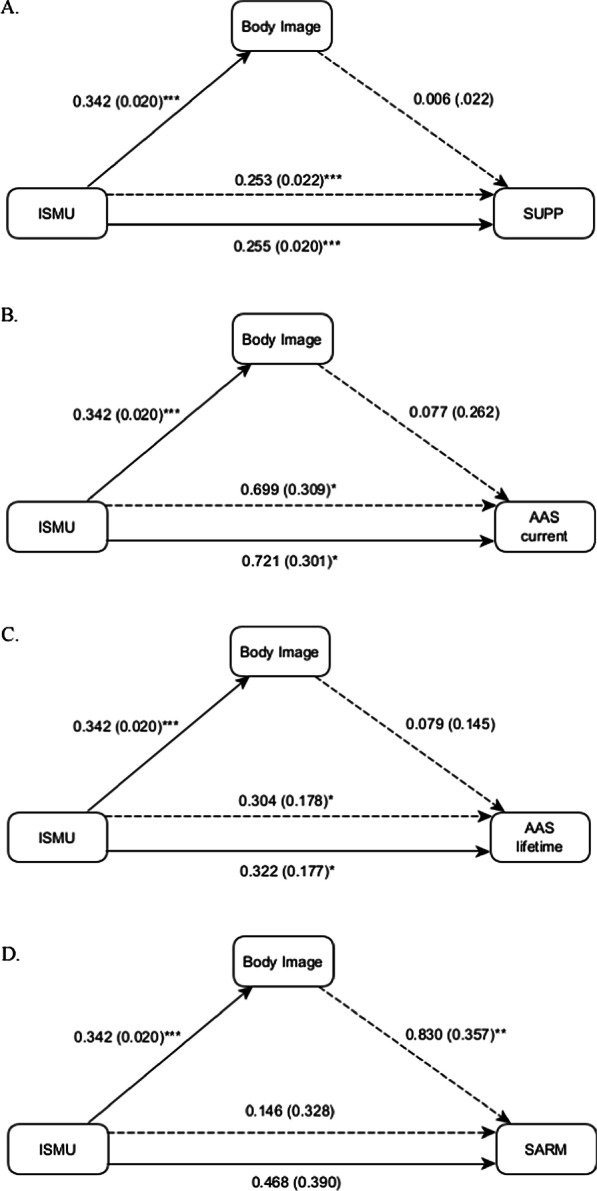
**a**–**d** Parameter estimates (and standard errors) of the mediation model with dietary supplement use (SUPP: **a**), current anabolic androgenic steroid use (AAS_current_: **b**), lifetime anabolic androgenic steroid use (AAS_lifetime_: **c**), and lifetime selective androgen receptor modulator use (SARM: **d**). ISMU: image-centric social media use. Dashed lines indicate parameter estimates that come from one single model with 2 predictors. Significance codes: *p < .05, **p < .01, ***p < .001

Figure 3a depicts the results of the mediation analyses with dependent variable supplement use. It shows that image-centric social media use has a significant total effect on supplement use (${b}_{ismu}=0.255, SE=0.020, t=12.6, p<.001$), indicating that for each standard deviation increase on the image-centric social media use scale, the score on the supplements scale increases with 0.255 standard deviations. Image-centric social media use also has a significant effect on body image (${b}_{ismu\to body image}=0.342, SE=0.020, t=17.3, p<.001$), indicating that the score on the body image scale increases with 0.342 standard deviations with each standard deviation increase on the image-centric social media use scale. In the model with body image and image-centric social media use, the effect of body image is not significant (${b}_{body image}^{*}=0.006, SE=0.022, t=0.278, p=0.340$). As a consequence, there is no significant mediation effect of body image on SUPP, and the indirect of 0.253 remains practically equal to the total effect of 0.255.

Figure 3b, c depicts the results of the mediation analysis with lifetime and current AAS as dependent variables. The total effect of image-centric social media use on lifetime AAS is significant in the directional test (${b}_{ismu}=0.322, SE=0.177, t=1.82, p=0.034$), indicating that the probability of AAS use increases with more image-centric social media use. To give an idea of the effects size, for the score of minus 1 standard deviation and plus 1 standard deviation from the mean on the image-centric social media use scale, the respective probability estimates of AAS use are 0.064 and 0.115. As in the previous model, however, the slope of body image is not significant (${b}_{body image}^{*}=0.079, SE=0.145, t=0.55, p=.24$), and therefore there is no evidence for a mediation effect of body image. The total effect of image-centric social media use on current AAS is slightly larger (${b}_{ismu}=0.699, SE=0.309, t=2.26, p=0.012$), but as for lifetime AAS there is no evidence for mediation.

Figure 3d depicts the results of the mediation analysis with SARM as dependent variable. The total effect of image-centric social media use on SARM is larger than its effect on AAS (${b}_{ismu}=0.468, SE=0.390, t=1.2, p=0.12$), yielding estimated probabilities of SARM use at minus 1 and plus 1 standard deviation from the mean on the image-centric social media use scale of, respectively, 0.016 and 0.039, but the effect is not statistically significant. The effect of body image on SARM is significant (${b}_{body image}^{*}=0.830, SE=0.357, t=2.33, p = 0.010$), yielding estimated probabilities of SARM use at minus 1 and plus 1 standard deviation from the mean on the body image scale of, respectively, 0.009 and 0.046, given a mean score of 0 on the image-centric social media use scale. As a consequence, there is a substantial difference between the total effect (0.468) and the indirect effect (0.148) of image-centric social media use. The difference between the total and direct effect cannot be attributed to the mediation effect of body image, since the total effect of image-centric social media use was not statistically significant.

## Discussion

The aim of the current study was to provide contemporary data on the use of dietary supplements, AAS and SARM, and test the idea that social media, and in particular its images, are associated with the use of these compounds in young male gym users as a result of a negative body image. Findings revealed that the larger part (83%) of young male gym users in the Netherlands uses dietary supplements (e.g., protein, creatine, etc.), and an estimated 9 versus 2.7% of this population have ever used AAS or SARM, respectively. In addition, image-centric social media use was positively associated with the use of dietary supplements and AAS, but not SARM. Furthermore, image-centric social media use was associated with body image, such that more of this type of social media use was related to a more negative body image. Body image, however, did not have a mediating impact on any of the relations between image-centric social media use and substance use.

### Prevalence of Dietary Supplement and Pre-workout Use

In a recent Dutch study among the general population, it was shown that 10% of the people aged 21–35 years use protein shakes, and only 1% use creatine [5]. This is in sharp contrast with our data revealing that 83% use dietary supplements, mainly protein (81%) and creatine (46%). This confirms our hypothesis that the use of ‘muscle-building’ supplements is highly prevalent among young male gym users. Our findings expand on previous data showing that 44% of Portuguese gym users use supplements, mostly protein (80%) and creatine (28%) [44], whereas 36.8% of Brazilian gym users use supplements, also mainly protein (38%) and creatine (8%) [3]. Of the participants in the present study that reported using supplements, 44% reported the use of pre-workout formulas, mainly to stimulate the motivation to work out and increase energy levels. This prevalence rate is considerably higher when compared to German regular gym users (12%) [45]. The reported prevalence reported in the present study can be considered worrying, as pre-workout formulas are associated with side-effects (dizziness and nausea) and adverse events, such as heart rhythm abnormalities [46]. In addition, a recent study, targeting the Dutch market, reported that pre-workout supplements, readily available in web shops, are ‘at risk’ of containing undeclared doping compounds, mostly anabolic steroids and stimulants [47]. In this regard, it is alarming that 25% of the pre-workout users in the present study use a pre-workout supplement before every workout.

### Prevalence of AAS Use

Our results show a current AAS prevalence rate of 3.6% and a life-time prevalence rate of 9% among young male gym users in the Netherlands. This is significantly higher than the 1% prevalence rate reported by an earlier study in the Netherlands, dating from 2013 [4]. However, the latter study included both male and female gym users from all ages, whereas the present work centered on young male gym users. In line with our results, a survey in the UK among 377 male and female gym users resulted in a current prevalence rate of 7%, albeit not assessed with randomized responses [6]. Simon and coworkers were the first to assess the use of doping compounds with randomized responses among German regular gym users and reported a life-time prevalence of 12.5% [48]. These results, however, may not be directly comparable to our results, as in the current study we assessed specifically the use of AAS and SARM, while Simon et al. assessed the use of doping substances in general. Further, a meta-analysis by Sagoe and coworkers indicated that the lifetime prevalence of AAS use in recreational sportspeople is 18.4% [21]. However, as the authors of the meta-analysis note, the validity of prevalence rates of the included studies is considerably limited, as most of them are not assessed by the RRT. Indeed, research with regular self-reports, so without RRT, on the use of doping substances has the potential to overestimate or underestimate prevalence rates [4, 21]. Hence, the prevalence data of the current study assessed with the RRT are a noteworthy addition to the literature.

Having a reliable idea of prevalence data of AAS use can be considered important, as the use of AAS is associated with health risks [15]. Although the acute risks of AAS use are relatively low [49], long-term effects of AAS include cardiac disease [13, 14], mood and anxiety disorders [50] and higher mortality risks [51]. Finally, it is worrisome that addiction to the use of AAS is highly prevalent [49, 52, 53], increasing the risk of health consequences in the long term.

### Prevalence of SARM Use

To the best of our knowledge, this is the first study to present prevalence rates (2.7%) of SARM use. It has been suggested that SARM use has been increased recently, although factual evidence has been lacking [18]. Users of SARM mainly claim that SARM are safe; however, hardly any clinical studies support the efficacy and safety of such experimental drugs [19]. In fact, in 2017 the US Food and Drug Administration (FDA) issued a public statement that SARM were being included in supplements and that these compounds posed an increased risk for heart attack, stroke, and liver damage [18]. The prevalence rates revealed by the current study give a call to medical doctors and other health care providers. That is, they should be aware of the increased popularity of SARM use and other appearance-related drugs among regular gym users. Medical complaints due to substance use may easily be overlooked in this group of non-professional athletes, particularly as individuals using these drugs emerge as sportive based on appearance, while factually substance use contributed to their muscular physiques.

### Body Dissatisfaction Among Young Male Gym Users

Based on the used body image scale, ~ 5% of the participants were not satisfied with their body. However, specified to muscularity dissatisfaction, ~ 20% of the participants were not satisfied with this body component. Previous work has revealed that body image concerns are associated with the use of dietary supplements in boys [54] and AAS in adult males [32, 34]. In line with these findings, the current study revealed a relationship between body image and the use of SARM, with a more negative body image being related to increased SARM use. However, in contrast to the prevailing literature [32, 34], the current study did not reveal a significant relationship between body image and the use of dietary supplements and AAS. In spite of the difficulty of explaining this unexpected finding, some speculations could be placed to provoke further thought on the association between body image and AAS use in men. Pope and coworkers have proposed that concerns about body image among young men, and subsequent AAS use, may be related to the emphasis on a muscular and lean physique in our current society [32]. In this regard, most research used *muscle dysmorphia* as a tool to assess body image, as opposed to *body image* in the current study. While muscle dysmorphia is a psychiatric disorder [55], body image only indicates the extent to which people were satisfied with their physical appearance. Although ~ 20% of the investigated population indicated that they were (partly) unsatisfied with their muscularity, this was not associated with the use of AAS. This may be partly explained by the population of recreational gym users included in the current study, as many studies on the relation between muscle dysmorphia and AAS use have been conducted among bodybuilders [56, 57]. In addition, the use of AAS for appearance-enhancing motives has become increasingly normalized in the Netherlands [58] and may just be a part of the gym culture, rather than the result of a negative body image. Such speculations obviously need to be corroborated by further research.

### The Role of Social Media in Body Image Concerns and Substance Use

Social media exposure has been associated with body image concerns in men [24], sexual minority men [59] and adolescents boys (11–18 years) [60]. In line, our results show a negative relationship between image-centric social media use and body image in young male gym users. The current study expands on previous findings by specifically showing that image-centric social media such as Instagram and exposure to fitness-related content, is associated with body dissatisfaction, and with the use of dietary supplements and AAS. Noteworthy is that the frequency of social media use did not show this association, indicating that particularly the content of social media is related to body dissatisfaction and the use of supplements and AAS. Image-centric social media use was not associated with the use of SARM. This non-significant relationship between image-centric social media use and SARM may be explained by the low prevalence rate of SARM.

So-called influencers on social media can have an enormous amount of followers, and are already part of companies’ marketing strategies [61, 62], because of their ability to affect the behavior of their followers [28]. These findings together show the potential influence of social media in young male’s decision making in the context of fitness, nutrition and doping.

### Clinical Relevance

Considering the high use of dietary supplements and pre-workout formulas, the considerable use of AAS and SARM, and the concerns regarding muscularity among young male gym users, it seems urgent to devote more attention to this population, particularly from the sports medicine community. This is particularly crucial in light of fitness currently being one of the most popular sports activities. Young male gym users are often highly motivated to reach their fitness goals, mostly an increase in muscle mass and strength in combination with a decrease in fat mass. Such goals are comparable to those of elite athletes. However, where elite athletes, a relative small population, are likely to receive high-quality advice from professionals such as strength and conditioning coaches, sports nutritionist and sports physicians, the relative large population of regular gym users has limited (free) access to such expert advice. Consequently, regular gym users mainly retrieve information on nutrition, supplements and training from the internet and social media [3]. Indeed, the present study shows that 65% use the internet and 32% use social media as their primary source of information on dietary supplements. In this regard, it is worrying that 38% of high-risk dietary supplements (e.g., pre-workout) sold online in the Netherlands contain undeclared doping compounds [47]. On top of that, it is alarming to note that regular gym users have a higher lifetime prevalence rate of AAS use than elite athletes [20]. Therefore, we call to action from national fitness associations, national anti-doping agencies and national sports medicine communities to work together to support gym users to reach their fitness goals in a healthy and sustainable manner. Furthermore, more attention should be given to the education of fitness professionals and healthcare providers about the use of compounds like dietary supplements, AAS and SARM, and associated body image concerns among gym users.

### Limitations and Recommendations

This study is innovative due to its large sample size, applying the RRT to estimate image and performance-enhancing drug use, including SARM use which can be considered as novel, and relating substance use to the impact of social media, particularly image-centric social media use. Despite these strengths, it is also important to acknowledge some limitations of the current study. First, data collection took place during the Covid-19 pandemic, which may have resulted in an underestimation of the AAS and SARM prevalence rates as a result of the closure of all gyms in the Netherlands preceding this study. Future studies should thus reproduce these prevalence rates. Also in relation to the link between image and performance-enhancing drugs use and social media, replications are desirable, as social media develops constantly and fast in rather unpredictable ways. Therefore, it seems important to keep track of social media trends, including its potential undesirable side effects.

Next, the present study may suggest that image-centric social media use is associated with body image and increased use of image and performance-enhancing drugs. It is important to note, though, that the correlational design impedes drawing inferences about any causal relationships among the variables. Future studies could omit this limitation by incorporating experimental, longitudinal or ecological momentary assessment designs to establish whether there exist any causal relationships, for example to assess if image-centric social media use impacts body image, or vice versa, if body image influences image-centric social media use. The potential future detection of such causal relations could subsequently aid in understanding the relation of these phenomena with the use of performance-enhancing drugs.

Furthermore, the present research relied on self-report measures to investigate social media use. Consequently, our findings pertain to self-perceptions of social media use, which inevitably adds bias, such as contingency of awareness of one’s social media habits and social desirability. Although the social media measures were based on previous research and scales revealed acceptable to good reliability, future research should further develop and validate scales to capture social media use. Particularly, the questionnaire measuring image-centric social media use should be developed in more detail as it relates to the comparison of physical appearance with others on social media in combination with the exposure to fitness-related content on social media. This deserves more validity research. Moreover, future researchers could explore the additional value of adding standardization of the time frame of reference as a valuable asset, such as how often people use image-centric social media on a typical day in terms of minutes or hours, to decrease potential variability in the current relative scores that capture the relative extent to which people use this type of social media. This is particularly important as the present findings seem to suggest that not so much the frequency of social media use may have undesirable effects, but rather the content of social media’s images relating to creating a perfect ‘me.’

## Conclusion

The larger part (83%) of young male gym users in the Netherlands use dietary supplements, mainly protein and creatine, and an estimated 9 and 3% of this population has ever used AAS or SARM, respectively. Image-centric social media use is positively associated with the use of dietary supplements and AAS among young male gym users, but this relationship was not mediated by body image.

## Supplementary Information

**Additional file 1.** Participants’ characteristics.

**Additional file 2.** Randomized response.

**Additional file 3.** Mediation analysis.

**Additional file 4.** Scale construction with Principal Component Analysis (PCA).

## Data Availability

All data supporting the results of this study may be made available from the corresponding author on reasonable request.
