# Supplementary material for: Social Media, Body Image and Resistance Training: Creating the Perfect ‘Me’ with Dietary Supplements, Anabolic Steroids and SARM’s
Source: Sports Med Open. 2021 Nov 10;7:81. doi: 10.1186/s40798-021-00371-1 (PMC8579410; doi:10.1186/s40798-021-00371-1)
Supplement: Supplementary file 1 — Additional file 1. Participants’ characteristics. [file 40798_2021_371_MOESM1_ESM.docx]

**Article title**: Social media, body image and resistance training: Creating the perfect ‘me’ with dietary supplements, anabolic steroids and SARM’s

**Journal name**: Sports Medicine - Open

**Authors:** Luuk Hilkens^1^, Maarten Cruyff^2^, Liesbeth Woertman^3^, Jeroen Benjamins^4, 5^, & Catharine Evers^4^

**Author affiliations:**

^1^ School of Sport and Exercise, HAN University of Applied Sciences, Nijmegen, The Netherlands

^2^ Department of Methodology & Statistics, Utrecht University, Utrecht, The Netherlands

^3^ Department of Clinical Psychology, Utrecht University, Utrecht, The Netherlands

^4^ Department of Social, Health, and Organizational Psychology, Utrecht University, Utrecht, The Netherlands

^5^ Department of Experimental Psychology, Helmholtz Institute, Utrecht University, Utrecht, The Netherlands

**Corresponding author**

Dr. Catharine Evers, Department of Social, Health, and Organizational Psychology, Utrecht University, PO Box 80140, 3508 TC Utrecht, The Netherlands, Email: [c.evers@uu.nl](mailto:c.evers@uu.nl)

**SUPPLEMENTAL FILE I: Participants’ characteristics**

*Nationality*

The majority of the participants was Dutch (97.1%), followed by ‘other’ (2.4%), Surinamese (0.2%), and Turkish (0.3%).

*Educational level*

The educational level of the participants was distributed as follows (from lower to higher level): preparatory secondary vocational education (3.5%), middle-level applied education (23.3%), senior general secondary education (22.5%), applied university-level education (20.4%), university preparatory education (13.5%), and university-level education (10.2%). Of the entire sample, almost half was employed (49%); a large part student (42%); and the remaining part self-employed (7%) or unemployed (2%).
