# Supplementary material for: Social Media, Body Image and Resistance Training: Creating the Perfect ‘Me’ with Dietary Supplements, Anabolic Steroids and SARM’s
Source: Sports Med Open. 2021 Nov 10;7:81. doi: 10.1186/s40798-021-00371-1 (PMC8579410; doi:10.1186/s40798-021-00371-1)
Supplement: Supplementary file 2 — Additional file 2. Randomized response. [file 40798_2021_371_MOESM2_ESM.docx]

**Article title**: Social media, body image and resistance training: Creating the perfect ‘me’ with dietary supplements, anabolic steroids and SARM’s

**Journal name**: Sports Medicine - Open

**Authors:** Luuk Hilkens^1^, Maarten Cruyff^2^, Liesbeth Woertman^3^, Jeroen Benjamins^4, 5^, & Catharine Evers^4^

**Author affiliations:**

^1^ School of Sport and Exercise, HAN University of Applied Sciences, Nijmegen, The Netherlands

^2^ Department of Methodology & Statistics, Utrecht University, Utrecht, The Netherlands

^3^ Department of Clinical Psychology, Utrecht University, Utrecht, The Netherlands

^4^ Department of Social, Health, and Organizational Psychology, Utrecht University, Utrecht, The Netherlands

^5^ Department of Experimental Psychology, Helmholtz Institute, Utrecht University, Utrecht, The Netherlands

**Corresponding author**

Dr. Catharine Evers, Department of Social, Health, and Organizational Psychology, Utrecht University, PO Box 80140, 3508 TC Utrecht, The Netherlands, Email: [c.evers@uu.nl](mailto:c.evers@uu.nl)

**SUPPLEMENTAL FILE 2: Randomized response**

There are different randomized response techniques (RRT). In the present study we used an adaptation of Kuk’s method (Kuk, 1990). Kuk’s method provides respondents with two decks of cards, a ‘yes’ deck and a ‘no’ deck. The ‘yes’ deck contains, for example, 5/6 red cards and 1/6 black cards, while the ‘no’ deck contains 1/6 red cards and 5/6 black cards. Respondents can draw a card from both decks and answer the sensitive question by naming the colour of the card from the deck that corresponds to the true answer. The advantage is that naming a colour as answer is more neutral than having to answer with a direct ‘yes’ or ‘no’ to the sensitive question.

In the present study we refrained from using colours as we were unable to control the colour settings of the different electronic devices that respondents used to respond to the questionnaire. Instead, we used geometric symbols for the two decks: a circle and a square. See Supplemental Figure 1 for an illustration.


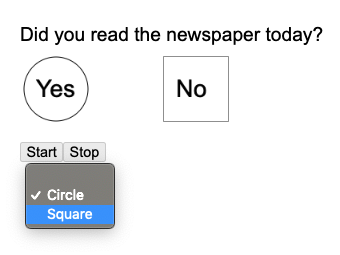


*Supplemental Figure 1. Illustration of a neutral practice question as part of the Randomized response technique to measure sensitive questions.*

After reading the sensitive question, respondents had to click a ‘start’ button, which resulted in the ‘yes’ and ‘no’ decks starting to alternate between the two geometric symbols with an interval of 100 ms. When the ‘stop’ button was hit, the alternation stopped, and the ‘yes’ appeared in the square and the ‘no’ in the circle, or vice versa. The ‘yes’ deck contained 5/6 circles and 1/6 squares, while the ‘no’ deck contained 1/6 circles and 5/6 squares. The respondent then answered the sensitive question by naming the geometric symbol from the deck that corresponded to the true answer.

To ensure a proper understanding of the answer mechanism, participants first received instructions on the procedure employed. To make participants familiar with the procedure, participants subsequently received two neutral practice questions: “Did you read the newspaper today?” and “Did you see the news today?”. During this practice trial, the ‘yes’ and ‘no’ decks contained 1/2 circles and 1/2 squares. Participants could repeat these practice questions as many times as necessary to understand the answer mechanism. All participants indicated to understand the procedure. Finally, the practice trial was followed by the three sensitive questions relating to substance use.
