# Supplementary material for: Social Media, Body Image and Resistance Training: Creating the Perfect ‘Me’ with Dietary Supplements, Anabolic Steroids and SARM’s
Source: Sports Med Open. 2021 Nov 10;7:81. doi: 10.1186/s40798-021-00371-1 (PMC8579410; doi:10.1186/s40798-021-00371-1)
Supplement: Supplementary file 3 — Additional file 3. Mediation analysis. [file 40798_2021_371_MOESM3_ESM.docx]

**Article title**: Social media, body image and resistance training: Creating the perfect ‘me’ with dietary supplements, anabolic steroids and SARM’s

**Journal name**: Sports Medicine - Open

**Authors:** Luuk Hilkens^1^, Maarten Cruyff^2^, Liesbeth Woertman^3^, Jeroen Benjamins^4, 5^, & Catharine Evers^4^

**Author affiliations:**

^1^ School of Sport and Exercise, HAN University of Applied Sciences, Nijmegen, The Netherlands

^2^ Department of Methodology & Statistics, Utrecht University, Utrecht, The Netherlands

^3^ Department of Clinical Psychology, Utrecht University, Utrecht, The Netherlands

^4^ Department of Social, Health, and Organizational Psychology, Utrecht University, Utrecht, The Netherlands

^5^ Department of Experimental Psychology, Helmholtz Institute, Utrecht University, Utrecht, The Netherlands

**Corresponding author**

Dr. Catharine Evers, Department of Social, Health, and Organizational Psychology, Utrecht University, PO Box 80140, 3508 TC Utrecht, The Netherlands, Email: [c.evers@uu.nl](mailto:c.evers@uu.nl)

**SUPPLEMENTAL FILE 3: Mediation analysis**

To test whether the effect of image-centric social media use on the respective dependent variables, i.e., supplements, AAS, and SARM, was mediated by body image, four separate mediation analyses were conducted. AAS use was separated for current use (last 12 months; AAS_current_) and lifetime use (AAS_lifetime_). For each mediation analysis four regression models were fitted to determine the total, the direct and the indirect effects of image-centric social media use (ismu). The total effects are measured by the slopes $b_{ismu}$ in the regression models with image-centric social media use as the sole predictor of the dependent variables. The direct effects are measured by the slope $b_{ismu}^{*}$ in regression models with both image centric social media use and body image as the predictors of the dependent variables. For the continuous dependent variable supplements use these are linear regression models, and for the three binary dependent variables AAS_current_, AAS_lifetime_ and SARM, these are logistic regression models accounting for randomized response [59]. The indirect (mediation) effects are measured by (the product of) the slopes and $b_{ismu\to body image}$ and $b_{body image}^{*}$. The former is the slope of image-centric social media use as predictor of body image, which is the same for all four dependent variables, and the latter is the slope of body image in the models with both image-centric social media use and body image as predictors of the dependent variables.

To establish whether there is a mediation effect of body image, the following hypotheses are tested. Given the expectation that image-centric social media use results in increased substance use, the significance of the total effects of image centric social media use are tested with the directional alternative hypothesis is $H_{A}:b_{ismu}>0$. The significance of the effect of image centric social media use on body image is tested with the directional hypothesis $H_{A}:b_{ismu\to body image}<0$, in line with the expectation that more image-centric social media use is associated with a less positive body image. The significance of the effect of body image on the dependent variables is tested with the directional hypothesis $H_{A}:b_{body image}^{*}<0$, in line with the expectation that a lower body image results in a higher substance use. Finally, the significance of the mediation effect is tested with the directional hypothesis $H_{A}:b_{body image}^{*}\cdot b_{ismu\to body image}>0$, the acceptance of which requires both slopes to be significant. However, this test is only valid for the models with supplements use as dependent variable, since these are all linear regression models. For the dependent variables AAS_current,_ AAS_lifetime_ and SARM, however, the slope $b_{body image}^{*}$ is measured on the logit scale, and therefore the product of $b_{ismu\to body image}$ and $b_{body image}^{*}$ is not meaningful. For these dependent variables, the significance of the mediation effects of body image on AAS and SARM is tested as the difference between the total and direct effect of image centric social media use according to the hypothesis $H_{A}:b_{ismu}{>b}_{ismu}^{*}$.
