# Supplementary material for: Social Media, Body Image and Resistance Training: Creating the Perfect ‘Me’ with Dietary Supplements, Anabolic Steroids and SARM’s
Source: Sports Med Open. 2021 Nov 10;7:81. doi: 10.1186/s40798-021-00371-1 (PMC8579410; doi:10.1186/s40798-021-00371-1)
Supplement: Supplementary file 4 — Additional file 4. Scale construction with Principal Component Analysis (PCA). [file 40798_2021_371_MOESM4_ESM.docx]

**Article title**: Social media, body image and resistance training: Creating the perfect ‘me’ with dietary supplements, anabolic steroids and SARM’s

**Journal name**: Sports Medicine - Open

**Authors:** Luuk Hilkens^1^, Maarten Cruyff^2^, Liesbeth Woertman^3^, Jeroen Benjamins^4, 5^, & Catharine Evers^4^

**Author affiliations:**

^1^ School of Sport and Exercise, HAN University of Applied Sciences, Nijmegen, The Netherlands

^2^ Department of Methodology & Statistics, Utrecht University, Utrecht, The Netherlands

^3^ Department of Clinical Psychology, Utrecht University, Utrecht, The Netherlands

^4^ Department of Social, Health, and Organizational Psychology, Utrecht University, Utrecht, The Netherlands

^5^ Department of Experimental Psychology, Helmholtz Institute, Utrecht University, Utrecht, The Netherlands

**Corresponding author**

Dr. Catharine Evers, Department of Social, Health, and Organizational Psychology, Utrecht University, PO Box 80140, 3508 TC Utrecht, The Netherlands, Email: [c.evers@uu.nl](mailto:c.evers@uu.nl)

**SUPPLEMENTAL FILE 4: Scale construction with Principal Component Analysis (PCA)**

**Table of Contents**

[Scale construction 3](#_Toc82777080)

[Image-centric Social Media Use (ISMU): 4](#_Toc82777081)

[Scree plot 4](#_Toc82777082)

[Component loadings and explained variance 4](#_Toc82777083)

[Frequency of social media use (FSMU): 6](#_Toc82777084)

[Scree plot 6](#_Toc82777085)

[Component loadings and explained variance 6](#_Toc82777086)

[DIETARY SUPPLEMENT USE (SUPP) 8](#_Toc82777087)

[Scree plot 8](#_Toc82777088)

[Component loadings and explained variance 8](#_Toc82777089)

[Scale statistics 10](#_Toc82777090)

[Histograms 10](#_Toc82777091)

[Correlations 10](#_Toc82777092)

# Scale construction

This document describes the construction of the scales image-centric social media use (ISMU), frequency of social media use (FSMU) and dietary supplement use (SUPP). The scale scores are obtained from a principal components analysis of the items covariance matrix. The scale scores are a weighted average of the item scores, with weights proportional to the component loadings of the 1st principal component. The scale scores are standardized with mean 0 and variance 1.

The principal components analyses are conducted in R with the function principal of the package psych.

For each scale the following results are displayed:

- the scree plot with eigenvalues (eigenvalues are proportional to explained variance)
- the factor loadings
- the (rescaled) proportion of explained variance by the 1st component

The section scale statistics presents:

- histograms of the scale scores
- correlations between the scales.

## Image-centric Social Media Use (ISMU):

- 8 items on 5-point Likert scales on
- amount of contact with fitness-related content on social media, and
- comparisons of participants’ physical appearance with peers and ‘influencers’.

### Scree plot


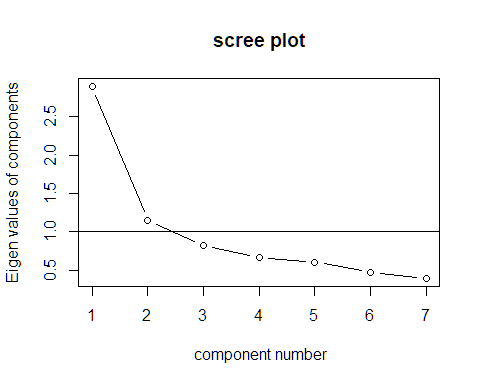


### Component loadings and explained variance

Loadings:
 PC1
Q44_1 0.756
Q44_2 0.873
Q45 0.495
Q46_1 0.401
Q46_2 0.704
Q46_3 0.760
Q46_4 0.626

 PC1
SS loadings 3.204
Proportion Var 0.458

Rescaled Proportion Variance Explained by PC1: 0.424

## Frequency of social media use (FSMU):

- two items on frequency of and time spent on checking social media

### Scree plot


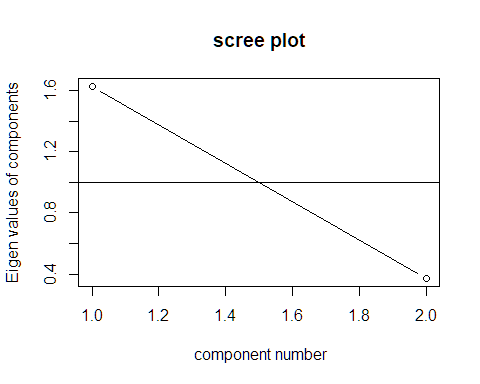


### Component loadings and explained variance

Loadings:
 PC1
Q41 1.051
Q42 1.408

 PC1
SS loadings 3.087
Proportion Var 1.543

Rescaled Proportion Variance Explained by PC1: 0.822

## DIETARY SUPPLEMENT USE (SUPP)

- 12 items for 12 different supplements
- 31-point scales measuring frequency of use

### Scree plot


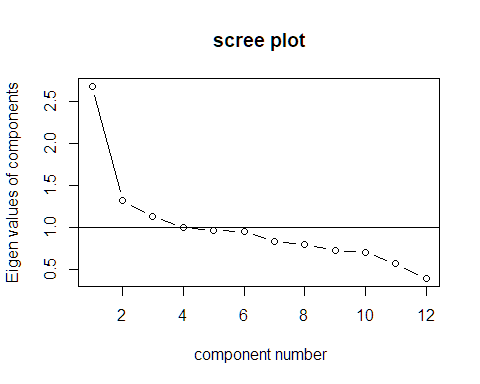


### Component loadings and explained variance

Loadings:
 PC1
Q56_1 7.742
Q56_2 1.934
Q56_3 1.036
Q56_4 0.504
Q56_5 11.558
Q56_6 6.886
Q56_7 2.879
Q56_8 0.739
Q56_9 0.307
Q56_10 0.427
Q56_11 1.900
Q56_12 1.000

 PC1
SS loadings 259.738
Proportion Var 21.645

Rescaled Proportion Variance Explained by PC1: 0.372

# Scale statistics

## Histograms


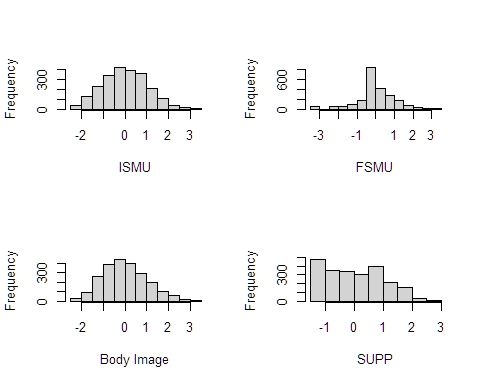


## Correlations

ISMU FSMU BI SUPP
ISMU 1.000 0.233 0.342 0.255
FSMU 0.233 1.000 0.134 0.093
BI 0.342 0.134 1.000 0.093
SUPP 0.255 0.093 0.093 1.000
